# Pharmacological interventions for delirium in intensive care patients: a protocol for an overview of reviews

**DOI:** 10.1186/s13643-016-0391-5

**Published:** 2016-12-07

**Authors:** Marija Barbateskovic, Laura Krone Larsen, Marie Oxenbøll-Collet, Janus Christian Jakobsen, Anders Perner, Jørn Wetterslev

**Affiliations:** 1 Copenhagen Trial Unit, Centre for Clinical Intervention Research, Rigshospitalet, Copenhagen University Hospital, Copenhagen, Denmark; 2 Centre for Research in Intensive Care, Rigshospitalet, Copenhagen University Hospital, Copenhagen, Denmark; 3 Department of neuroanaesthesiology, Rigshospitalet, Copenhagen University Hospital, Copenhagen, Denmark; 4 Department of Intensive Care, Rigshospitalet, Copenhagen University Hospital, Copenhagen, Denmark; 5 Department of Cardiology, Holbaek Hospital, Holbaek, Denmark

**Keywords:** Delirium, Prevention, Management, Antipsychotics, Sedatives, Systematic review, Intensive care unit, Critically ill, Pharmacological interventions

## Abstract

**Background:**

The prevalence of delirium in intensive care unit (ICU) patients is high. Delirium has been associated with morbidity and mortality including more ventilator days, longer ICU stay, increased long-term mortality and cognitive impairment. Thus, the burden of delirium for patients, relatives and societies is considerable.

Today, reviews of randomised clinical trials are produced in large scales sometimes making it difficult to get an overview of the available evidence. A preliminary search identified several reviews investigating the effects of pharmacological interventions for the management and prevention of delirium in ICU patients. The conclusions of the reviews showed conflicting results. Despite this unclear evidence, antipsychotics, in particular, haloperidol is often the recommended pharmacological intervention for delirium in ICU patients.

The objective of this overview of reviews is to critically assess the evidence of reviews of randomised clinical trials on the effect of pharmacological management and prevention of delirium in ICU patients.

**Methods/design:**

We will search for reviews in the following databases: Cochrane Library, MEDLINE, EMBASE, Science Citation Index, BIOSIS, Cumulative Index to Nursing and Allied Health Literature, Latin American and Caribbean Health Sciences Literature, and Allied and Complementary Medicine Database.

Two authors will independently select references for inclusion using Covidence, extract data and assess the methodological quality of the included systematic reviews using the ROBIS tool. Any disagreement will be resolved by consensus.

We will present the data as a narrative synthesis and summarise the main results of the included reviews. In addition, we will present an overview of the bias risk assessment of the systematic reviews.

**Discussion:**

Results of this overview may establish a way forward to find and update or to design a high quality systematic review assessing the effects of the most promising pharmacological intervention for delirium in ICU patients.

**Systematic review registration:**

PROSPERO - CRD42016046628.

**Electronic supplementary material:**

The online version of this article (doi:10.1186/s13643-016-0391-5) contains supplementary material, which is available to authorized users.

## Background

### Description of the condition

Delirium is a complex acute organic syndrome characterised by a reduced ability to focus, sustain, or shift attention, and either a change in cognition (memory deficits, disorientation, or language disturbance) or the development of a perceptual disturbance (hallucinations or delusions) [1]. The pathophysiology of delirium is superficially understood, and the mechanisms involved are likely to be multiple [2, 3]. Delirium has been classified into motoric subtypes: (1) hypoactive delirium (pure lethargy, withdrawal, flat affect, apathy and decreased responsiveness), (2) hyperactive delirium (agitation, restlessness, attempting to remove catheters and emotional lability), and (3) a mixed form delirium (fluctuation between hypoactive and hyperactive delirium) [4].

The prevalence of delirium is highest in hospitalised older individuals and varies depending on the patient characteristics, setting of care, and sensitivity of the detection method. In the community, the overall prevalence of delirium is low (1 to 2%) but increases with age, rising to 14% in individuals older than 85 years [5]. In nursing homes and residential homes, the prevalence of delirium is 8 to 9% [6]. At the time of hospital admission, the prevalence of delirium is an estimated 14 to 24% and the incidence 6 to 56% [7]. Delirium occurs in 9 to 87% of individuals postoperatively [8], in 40 to 60% of the spontaneously breathing patients in intensive care, in 60 to 89% of the mechanically ventilated patients in intensive care [9], and up to 83% of all individuals at the end of life [10].

Delirium is increasingly being recognised as a significant contributor to the morbidity and mortality of the intensive care unit (ICU) patients. Studies have shown an increase in total ventilator days, ICU length of stay, and long-term cognitive impairment [11, 12]. Pandharipande et al. [11] followed 821 surgical and medical ICU patients and found that longer duration of delirium was an independent risk factor for worse global cognition scores at both 3 and 12 months after discharge. Ely et al. [12] evaluated a smaller cohort of 224 ICU patients, and their results showed that delirium was independently associated with 3.2 (95% Cl 1.4–7.7; *P* = .008) increased risk of 6-month mortality. Contrary to this, in a larger cohort of 1112 ICU patients, Klouwenberg et al. [13] did not find an association between delirium and mortality after adjusting for changes in disease severity before the onset of delirium. Delirium complicates hospital stay for at least 20% of the 12.5 million patients 65 years of age or older who are hospitalised each year and increases hospital costs by $2500 per patient. An estimated $6.9 billion of medical hospital expenditures are attributable to delirium [5]. Substantial additional costs accumulate after hospital discharge because of the need for institutionalisation and rehabilitation services. In the ICU, within the mechanically ventilated patients, delirium has been associated with 39% higher intensive care costs and 31% higher hospital costs [14].

Different tools exist for the assessment of delirium in the ICU. The Confusion Assessment Method for the ICU (CAM-ICU) and the Intensive Care Delirium Screening Checklist (ICDSC) are often used [15]. Studies have indicated that delirium might be undetected in more than 65% of the ICU patients when comparing clinical identification of the presence of delirium to identification by the CAM-ICU or ICDSC scores [16, 17]. Hypoactive and mixed form delirium are the most common within the ICU patients [18, 19], and hypoactive delirium has been suggested to be more deleterious than the mixed form with worse outcomes and more long-term harmful effects [4].

### Description of the interventions

Abnormalities in cerebral oxidative metabolism, direct neurotoxic effects of inflammatory cytokine alterations in neurotransmitters (e.g. cholinergic, dopaminergic, serotonergic), and gamma-aminobutyric pathways are all believed to be involved in the pathophysiology of delirium [20]. Pharmacological interventions for delirium have focused on alterations in neurotransmitter pathways, in particular, dopaminergic and cholinergic pathways.

Several pharmacological strategies for delirium in the ICU patients have been investigated:

1. Antipsychotics (both typical antipsychotics e.g. haloperidol and atypical antipsychotics e.g. olanzapine, quetiapine, risperidone, aripiprazol, and ziprasidone)
2. Sedatives (e.g. benzodiazepines (midazolam, lorazepam, diazepam), propofol, and dexmedetomidine (alpha-2-antagonist))
3. Cholinesterase inhibitors (rivastigmine)
4. Opioids (e.g. morphine)
5. Melatonine and melatonine antagonists (e.g. ramelteon)

### Guidelines and recommendations

Examples of guidelines and recommendations for delirium in the ICU patients:

- The Society of Critical Care Medicine does not recommend haloperidol for the management of delirium due to lack of evidence, and rivastigmine is also not recommended due to an increased risk of torsades de pointes. Dexmedetomidine rather than benzodiazepine is recommended for sedation to reduce delirium in patients when the delirious symptoms are unrelated to alcohol and benzodiazepine withdrawal. Early mobilisation of patients is recommended to reduce incidence and duration of delirium. The Society of Critical Care Medicine recommends not using pharmacologic delirium prevention protocols (including the use of haloperidol, atypical antipsychotics and dexmedetomidine) [21].
- The Intensive Care Society in the UK recommends management of delirium with haloperidol, where no organic cause can be identified and treated. Olanzapine may be used as an alternative to haloperidol. Chlorpromazine, risperidone, and benzodiazepines are not recommended. Non-pharmacological interventions for the prevention of delirium are recommended. These interventions include providing psychological support and orientation (communication), stable environment (lights, noise, temperature), maintaining competences (e.g. correct sensory impairments, maintain activity level), and removing potential organic drivers by identifying and treating organic causes [22].
- The German guidelines for the management of analgesia, sedation and delirium in intensive care recommend management of delirium with haloperidol, risperidone or olanzapine. Low-dose haloperidol may be used prophylactically in geriatric delirious patients [23].
- The Danish Society of Anaesthesiology and Intensive Care states that for the management of manifest delirium, it is a medical judgement whether a pharmacological drug should be given, even though it is noted that no evidence for using pharmacological management exists. In these circumstances, olanzapine or haloperidol may be administered. Benzodiazepines may be added if the patient is still agitated and sedation with dexmedetomidine may be administered as a last resort. In the recommendations by The Danish Society of Anaesthesiology and Intensive Care, it is mentioned that prophylactic-administered haloperidol and ziprasidone does probably not reduce the duration of delirium in mechanically ventilated patients, but a precise advice against the use is not given [24].

For delirium, in general, haloperidol is recommended for the management of delirium patients with severe agitation or those who are considered a risk to themselves or to others. Prevention strategies do not include prophylactic medication [1, 25–27].

### How the interventions might work

Given the multiple mechanisms possibly resulting in the development of delirium, a range of pharmacological drugs have been studied. These different drugs target the suspected neurotransmitter or receptor imbalances, which may cause the distressing cognitive symptoms, hallucinations, risky physical behaviour, etc.

In the following, we describe the theoretical therapeutic rationale for each type of drug.

#### Antipsychotics (dopaminergic pathway)

The therapeutic effects of antipsychotics in delirium may be mediated via a number of different mechanisms that include sedative or anxiolytic effects, antipsychotic actions, or direct effects upon the proposed core neurochemical disturbances of delirium [28].

#### Sedatives

The benzodiazepines midazolam and lorazepam (and to a lesser extent, diazepam), propofol and dexmedetomidine are the commonly used sedatives within the ICU [29]. The opioid remifentanil is also used because of its sedative effects. Benzodiazepines may act through g-aminobutyric acid type A (GABAA) receptors, as in part does propofol, whereas dexmedetomidine is an a2-adrenoceptor agonist, and remifentanil is a μ-opioid receptor agonist [3].

#### Cholinesterase inhibitors

Impaired cholinergic neurotransmission seems to have an important role in the development of delirium. In patients with delirium, serum anticholinergic activity has been found to be increased [30] and, particularly in elderly patients, drugs with anticholinergic effects can cause delirium [31].

#### Opioids

Unrelieved pain induces stress responses characterised by tachycardia, increased myocardial oxygen consumption, hyper-coagulability, immunosuppression, and persistent catabolism. Ensuring adequate pain control might be essential. At low doses, opioids provide analgesia and in high doses, they provide anxiolysis and sedation [32]. The mechanism of action of pain management with ketamine, a N-methyl-D-aspartate receptor blocker, is not well understood.

#### Melatonine

Melatonine is a hormone normally produced in the pineal gland. It is assumed to help regulate sleep-wake cycles or the circadian rhythm. Production of melatonine is stimulated by darkness and inhibited by light. Patients admitted to the ICU develop impaired sleep patterns. Factors which contribute to the sleep impairment include the use of opioids and benzodiazepines, which disrupt rapid eye movement (REM), the impact of specific patient therapies such as asynchrony to mechanical ventilation, arousal related to patient care-related activities, environmental noise, and non-phasic light exposure [33]. Lower levels of melatonine and disrupted circadian release of melatonine have been associated with ICU delirium [34].

### Why it is important to do this overview

In the critically ill patients, 40 to 89% are reported to be affected by delirium and poor clinical outcomes are associated with the syndrome [9, 35–38]. Delirium patients may experience functional decline and long-term cognitive impairment [39–41]. Furthermore, delirium is associated with increased morbidity, lengthened duration of mechanical ventilation, ICU and hospital lengths of stay, and mortality [12, 14, 36, 39–43].

The economic burden of delirium is significant [44]. In hospitals, ICU beds are expensive, and some countries spend 0.7% of the national gross product on intensive care [44]. Due to an aging population and the increasingly advanced medical and surgical care, the number of patients in need of intensive care is expected to increase in the coming years [45].

### The evidence on the effects of pharmaceutical interventions

A number of trials investigating pharmacological prevention or management options have been published. However, uncertainty regarding the benefits and harms of pharmacological interventions, remains considerable, and trials have shown either positive [46, 47], equipoise [48, 49], or negative results [50].

Trials investigating the effects of antipsychotics (quetiapine, ziprazidone, risperiodene, haloperidol) have been conducted [46, 48, 51, 52]. The use of antipsychotics is associated with neurological side effects, including the development of extrapyramidal side effects, tardive dyskinesia, and neuroleptic malignant syndrome [1]. Management of delirium with haloperidol has been found to lengthen the QT interval, which can lead to torsades de pointes tachycardia that can degenerate to ventricular fibrillation and sudden death [53].

No sedative agent has been reported to improve survival in randomised clinical trials, despite at least 90 trials comparing sedative regimens in a systematic review [54]. In another meta-analysis comparing propofol to any sedative agent, propofol did not show to reduce mortality but may result in a reduction in the length of stay in the ICU when compared to benzodiazepines, but not when compared to midazolam [55]. Dexmedetomidine may have advantages compared with benzodiazepines, since it produces analgesia, causes less respiratory distress, and provides a different type of sedation in which patients are more interactive and so their needs are potentially easier communicated [56]. Dexmedetomidine compared to lorazepam and midazolam in randomised clinical trials has been shown to result in less delirium and a shorter duration of mechanical ventilation [56–58].

Morphine has been compared with haloperidol in the management of hyperactive delirium and was found to have a quick beneficial effect and no adverse effects [59]. Ketamine has been compared to placebo in the prevention of delirium and was found to lower the incidence of delirium [60].

Rivastigmine has in one trial been compared to placebo for the management of delirium, as an adjunct to usual care based on haloperidol. The trial was stopped early due to increased mortality in the rivastigmine group; thus, rivastigmine is not recommended to manage delirium in the ICU [50].

### Objectives

The objective of this overview of all reviews and meta-analyses is to critically assess the evidence of reviews on the effects of pharmacological management and prevention of delirium in intensive care patients.

## Methods

This protocol was written in accordance with the Preferred Reporting Items for Systematic Review and Meta-Analysis Protocols checklist (Additional file 1). Systematic review methods will follow the principles outlined in the Cochrane Handbook [61] and the recommendations given by Robinson et al. [62].

The protocol is registered on the PROSPERO database (CRD42016046628).

### Criteria for considering reviews for inclusion

All reviews and meta-analyses of pharmacological interventions for managing or preventing delirium in ICU patients that fulfil the following criteria will be eligible for inclusion:

### Types of studies

We will include all reviews and meta-analyses. We will define a systematic review as a review positively fulfilling the checklist in Preferred Reporting Items for Systematic Reviews and Meta-Analyses (PRISMA) [63].

### Participants

We will include ICU patients defined as patients treated in an ICU of any specialty (e.g. medical, surgical, trauma, cardiac) or similar terms according to definitions applied by the review authors. We will include reviews of any ICU patients equal to 18 years or older. In addition, we will include elective cardiac surgical population and acutely operated patients.

Reviews on the following patients will be excluded: ICU patients with delirium caused by alcohol withdrawal, terminally ill patients, patients admitted to the emergency care department, and elective surgical patients in general.

Reviews including ICU patients as well as other patient groups are eligible for inclusion if the ICU patients are described or analysed separately or the results/tables are given separately for this group of patients. In addition, if a review has included both randomised clinical trials and observational studies, the systematic review will only be eligible for inclusion in the analysis if the results of the randomised clinical trials are presented separately.

### Interventions

All pharmacological interventions both for the management and the prevention of delirium will be accepted. We will accept all reviews comparing a pharmacological agent with the following: placebo, any other pharmacological agent, or combinations of pharmacological and non-pharmacological interventions (bundle). Complementary therapies are eligible for inclusion too. We will also accept reviews if they are just describing pharmacological agents or combinations of these.

### Outcomes of interest

Results on all primary and secondary outcomes of the included systematic reviews will be reported. However, we will define primary and secondary outcomes for this overview as follows:

Primary outcomes

1. All-cause mortality
2. Proportion of participants with a serious adverse event defined as any untoward medical occurrence that resulted in death, was life threatening, was persistent, led to significant disability, jeopardised the participant, led to hospitalisation, or prolonged hospitalisation [64]
3. Proportion of participants with resolution of delirium symptom at end of treatment (as defined by review authors) (RR) and proportion of participants with delirium despite the administration of pharmacological drug before diagnosing delirium (prevention) (RR)

Secondary outcomes

1. Quality of life at end of treatment (any valid continuous quality of life scale used by the review authors)
2. Proportion of participants with non-serious adverse events, defined as any non-serious adverse event. Each adverse event will be analysed separately.
3. Cognitive function (any valid continuous cognitive function scale used by the review authors)

Exploratory outcomes

1. Duration of ICU stay
2. Duration of mechanical ventilation

The assessment time point of primary interest will be the time point closest to 3 months after randomisation. We will secondly assess each outcome at maximum follow-up.

## Search methods for identification of reviews

### Electronic searches

Reviews that fulfil the inclusions criteria will be identified through literature searching specific designed to identify relevant reviews.

The following databases will be searched:

- Cochrane Library
- MEDLINE (OvidSP)
- EMBASE (OvidSP)
- Science Citation Index-Expanded (Web of Science)
- BIOSIS Previews (Web of Science)
- Cumulative Index to Nursing and Allied Health Literature (CINAHL)
- Latin American Caribbean Health Sciences Literature (LILACS)
- Allied and Complementary Medicine Database (AMED) (Additional file 2)

### Searching other sources

Reference lists of reviews, relevant papers, randomised and non-randomised trials as well as editorials will be screened manually for potentially includable reviews.

### Data collection and analysis

The following methods on data collection and data analyses will be used.

### Selection of reviews

Two authors (MB, LKL/MO) will independently screen the titles and abstracts of all reports identified by the searches using the systematic review management toll Covidence [65]. Reports which are deemed potentially relevant by any of the reviewers will be obtained in full text, and the same two reviewers will independently assess these for inclusion. Any disagreement will be resolved by consensus.

### Data extraction and management

Two authors (MB, LKL/MO) will independently extract predefined data of the included reviews using a data extraction form (Additional file 3) which is specifically designed and piloted by the review team. Any disagreement concerning the extracted data will be discussed between the authors (MB, LKL/MO). If no agreement can be reached, a third author (JCJ/JW) will resolve the issue. Whenever necessary, corresponding authors will be contacted to clarify issues related to data reporting or if further review details are needed.

### Assessment of methodological quality of included reviews

The methodological quality of the included reviews will be assessed independently by two authors (MB, LKL/MO). First, all reviews will be checked against the PRISMA statements [63]. Hereafter, we will assess the methodological quality of the reviews fulfilling the PRISMA statements using the ROBIS tool [66]. Disagreements concerning the methodological assessment will be discussed between the authors and a third author (JCJ/JW) will be involved if no agreement can be reached.

We will assess risk of bias by assessing four domains, which cover key review processes: study eligibility criteria, identification and selection of studies, data collection and study appraisal, and synthesis and findings. Each domain will be assessed for information used to support the judgement, signalling questions, and judgement of concern about risk of bias. The signalling questions will be answered “Yes”, “Probably Yes”, “Probably No”, “No” and “No Information”, with “Yes” indicating low concerns. Hereafter each bias domain is judged as “Low”, “High”, or “Unclear”. For a domain to be judged low level of concern, then all signalling questions for the domain are Yes or Probably Yes. Concern about bias will be raised if any signalling questions is answered “No” or “Probably No” [67].

The non-systematic reviews will be assessed as high risk of bias notwithstanding that the methodological quality of these may not be low according to other standards.

Our conclusions will primarily be based on the systematic reviews assessed with low risk of bias according to ROBIS [66].

### Data synthesis

We will categorise reviews into the following:

1. Systematic reviews according to PRISMA with low risk of bias assessed with ROBIS
2. Systematic reviews according to PRISMA with high risk of bias assessed with ROBIS
3. Non-systematic reviews according to PRISMA

We will present the data as a narrative synthesis and summarise the main results of the included reviews. In addition, we will present an overview of the bias risk assessment of the assessed systematic reviews.

For systematic reviews deemed to be low risk of bias, we will reassess risk of bias in the included trials. For each identified intervention we will, if possible, perform meta-analysis and trial sequential analysis [68] of the low risk of bias trials.

### Assessment of risk of bias of studies included in systematic reviews deemed to be low risk of bias

MB and LKL/MO will independently assess the methodological quality of each included trial, defined by the design of the trial and reporting. Any disagreement will be discussed between the authors. We will assess the risk of bias according to the Cochrane Handbook of Systematic Reviews of Interventions [61].

For all included trials, we will assess the following risk of bias domains: random sequence generation, allocation sequence concealment, blinding of participants and personnel, blinding of outcome assessment, incomplete outcome data, selective outcome reporting, other bias risk and overall risk of bias. Based on this assessment, the included trials and each outcome result will be defined as low risk of bias if all bias domains are judged as low risk of bias.

Classification will be made according to the domains below:

#### Random sequence generation

- Low risk: If sequence generation is achieved using computer, random number generator or a random numbers table. Drawing lots, tossing a coin, shuffling cards and throwing dice are also being considered adequate if performed by an independent adjudicator.
- Unclear risk: If the method of randomization is not specified.
- High risk: If the allocation sequence is not random.

#### Allocation sequence concealment

- Low risk: If the allocation of patients is performed by a central independent unit, on-site locked computer, identically looking numbered sealed opaque envelopes, and drug bottles or containers prepared by an independent investigator. There must be no risk of the investigator knowing the sequence.
- Unclear risk: If the trial is classified as randomised but the allocation concealment process is not described.
- High risk: If the allocation sequence is known to the investigators who assigned participants.

#### Blinding of participants and personnel

- Low risk: If the participants and the personnel are blinded to treatment allocation and this is described.
- Unclear risk: If the procedure of blinding is insufficiently described or not described at all.
- High risk: If blinding of participants and personnel is not performed.

#### Blinding of outcome assessment

- Low risk: If the trial investigators performing the outcome assessments, analyses and calculations are blinded to the intervention.
- Unclear risk: If the procedure of blinding is insufficiently described or not described at all.
- High risk: If blinding of outcome assessment is not performed.

#### Incomplete outcome data

- Low risk: (1) There are no dropouts or withdrawals for all outcomes or (2) the numbers and reasons for the withdrawals and dropouts for all outcomes are clearly stated and can be described as being similar in both groups. As a general rule, the trial is judged as at a low risk of bias due to incomplete outcome data if the number of dropouts is less than 5%. However, the 5% cut off is not definitive.
- Unclear risk: The numbers and reasons for withdrawals and dropouts are not clearly stated.
- High risk: The pattern of dropouts can be described as being different in the two intervention groups or the trial uses improper methodology in dealing with the missing data, e.g. last observation carried forward.

#### Selective outcome reporting

- Low risk: A protocol is published before or at the time the trial is begun, and the outcome called for in the protocol is reported on.
- Unclear risk: If there is no protocol and the outcome is not reported on.
- High risk: If the outcomes which are called on in a protocol are not reported on.

#### Other bias risk

- Low risk of bias: The trial appears to be free of other components (for example, academic bias or for-profit bias) that could put it at risk of bias.
- Unclear risk of bias: The trial may or may not be free of other components that could put it at risk of bias.
- High risk of bias: There are other factors in the trial that could put it at risk of bias (for example, authors have conducted trials on the same topic, for-profit bias)

#### Overall risk of bias

We will classify all trials as follows:

- Overall low risk of bias: The trial will be classified as overall “low risk of bias” only if all of the bias domains described in the above paragraphs are classified as “low risk of bias”.
- Overall high risk of bias: The trial will be classified “high risk of bias” if any of the bias risk domains described in the above are classified as “unclear” or “high risk of bias”.

In addition, we will assess the domains “Blinding of outcome assessment”, “Incomplete outcome data”, and “Selective outcome reporting” for each outcome. Thus, we will be able to assess the bias risk for each result.

We will prepare a summary assessment of the risk of bias across trials and for each important outcome (across domains) by preparing a “Risk of bias graph” and a “Risk of bias summary figure” [61].

### Measures of treatment effect

Risk ratio (RR) with 95% confidence interval (Cl) and TSA adjusted CI will be calculated for dichotomous outcomes. For continuous outcomes, both end-scores and change scores will be included in the analyses. End-scores will be used if both are reported. Mean difference (MD) and standardised mean difference (SMD) with 95% Cls and TSA adjusted Cls will be calculated for continuous outcomes.

We have defined three co-primary outcomes and three secondary outcomes; thus, we will also report Cl’s as Cl 97.5%.

### Unit of analysis issues

We will only include participants according to the treatment group of the randomised clinical trials, therefore, no unit of analysis issues.

### Dealing with missing data

We will contact trial investigators of the original report for important missing data.

For both dichotomous and continuous outcomes, we will not be imputing missing data for any outcomes in the primary analysis and intention-to-treat data will not be used if the original report did not contain such data.

If standard deviations (SD) are not reported, the SDs will be calculated using data from the trial if possible.

In the sensitivity analysis for dichotomous and continuous outcomes, imputed data will be used, see “Sensitivity analysis”.

### Assessment of heterogeneity

We will assess signs of heterogeneity by visual inspection of the forest plots.

We will assess presence of statistical heterogeneity by chi-squared test with significance set at *P* < 0.10 and by measuring the quantities of heterogeneity by *I* ^2^ statistic [69]. We will consider *I* ^2^ of 40% or higher as substantial heterogeneity.

We will explore potential clinical heterogeneity by conducting the prespecified subgroup analyses, see “Subgroup analysis and investigation of heterogeneity”.

### Assessment of reporting biases

We will visually assess funnel plots for signs of asymmetry if 10 or more trials are included in an analysis [61, 70].

We will test asymmetry within dichotomous outcomes with the Harbord test [71] and for continuous outcomes regression asymmetry test [72]. Adjusted rank correlation will be used [73].

### Data synthesis

#### Meta-analysis

We will perform meta-analysis of outcomes with comparable effect measures where more than one trial is included. We will not meta-analyse trials if the clinical heterogeneity is substantial. The statistical software Review Manager [74] provided by the Cochrane Collaboration and the TSA [75] software ver. 0.9 CTU will be used to meta-analyse data.

#### Assessment of significance

We will assess our intervention effects with both random-effects model meta-analyses (67–69) and fixed-effect model meta-analyses [76, 77]. Anticipating large clinical as well as statistical heterogeneity, we will generally prefer reporting the result from a random-effects model. However, if one or two trials dominate the acquired evidence (e.g. with more than 80% of the randomised patients [78–80] the random-effects model may grossly overestimate the intervention effect and in this situation, we will report primarily the result from the fixed-effect model. That is, we will primarily report the result from the model with the most conservative point estimate of the two [70], being the estimate closest to zero effect. If the two estimates are approximately equal, we will use the estimate with the widest CI.

We use three primary outcomes and, therefore, we will consider a *P* value of 0.025 or less as statistically significant analysing the primary outcomes [70, 81]. We use three secondary outcomes and, therefore, we will consider a *P* value of 0.025 or less as statistically significant analysing the secondary outcomes [70]. We will use the eight-step procedure to assess if the thresholds for significance are crossed [70].

#### Trial sequential analysis

Cumulative meta-analyses are at risk of producing random errors due to sparse data and multiple testing of accumulating data [68, 82–89]; therefore, TSA [75] can be applied to assess this risk [90]. The required information size and the required number of trials [91] (which is the number of participants and trials needed in a meta-analysis to detect or reject an a priori prespecified realistic intervention effect) can be calculated in order to minimise random errors [92]. The required information size takes into account the event proportion in the control group, the assumption of a plausible relative risk (RR) reduction, and the heterogeneity variance [93] of the meta-analysis [92]. TSA enables testing for significance to be conducted each time a new trial is included in the meta-analysis. On the basis of the required information size and the required number of trials, trial sequential monitoring boundaries can be constructed. This enables one to determine the statistical inference concerning cumulative meta-analysis that has not yet reached the required information size [68, 85, 86, 88].

Firm evidence for benefit or harms may be established if the trial sequential monitoring boundary is crossed before reaching the required information size; in which case, further trials may turn out to be superfluous. In contrast, if the boundary is not surpassed, one may conclude that it is necessary to continue with further trials before a certain intervention effect can be detected or rejected. Firm evidence for lack of the postulated intervention effect can also be assessed with TSA. This occurs when the cumulative Z-score crosses the trial sequential monitoring boundaries for futility.

We will use relatively conservative estimations of the anticipated intervention effect estimates to reduce the risk of random error [70]. Large anticipated intervention effects lead to small required information sizes and the thresholds for significance will be less strict after the information size has been reached [70].

We will analyse all primary and secondary outcomes with TSA. We will estimate the diversity-adjusted required information size [92] based on the proportion of patients with an outcome in the control group. In addition, we will use a family-wise error rate (FWER) of 5% [70] leading to a statistical significance level of 3.3% for each of the co-primary outcomes, a beta of 20%, and a diversity (*D* ^2^) [92] suggested by the trials in the meta-analysis [70]. As a sensitivity analysis, we will use a diversity of 20% if the actual measured heterogeneity is in fact zero because in this case heterogeneity will most likely increase when further trials are added until the required information size is reached. As anticipated intervention effects for the primary and secondary outcomes in the trial sequential analysis, we will use realistic a priori relative risk reductions (RRR) or increases of 20% RRR or a 20% relative risk increase (RRI). Furthermore, we will use a RRR or a RRI based on the confidence limit closest to null effect in the 96.7% Cl in the traditional meta-analysis.

### Subgroup analysis and investigation of heterogeneity

Subgroup analyses will be performed to seek to determine if the efficacy and safety of the treatment options are influenced by the types of ICU populations.

The following subgroup analyses will be conducted if data permit:

- Trials with overall high risk of bias compared to trials with overall low or uncertain risk of bias

According to population

- Medical patients
- Surgical patients
- Cardiac surgical patients
- Acutely operated patients

### Sensitivity analysis

To assess the potential impact of bias, a sensitivity analysis for each outcome will be performed where trials with overall “high risk of bias” will be excluded.

To assess the potential impact of the missing data for dichotomous outcomes, the two following analyses will be performed:

1. “Best-worst-case” scenario: It will be assumed that all participants lost to follow-up in the experimental group survived, had no serious adverse event, and had no morbidity; and all those with missing outcomes in the control group did not survive, had a serious adverse event, and had morbidity.
2. “Worst-best-case” scenario: It will be assumed that all participants lost to follow-up in the experimental group did not survive, had a serious adverse event, and had morbidity; and all those with missing outcomes in the control group did survive, had no serious adverse event, and had no morbidity.

Results from both scenarios will be presented in the review.

To assess the potential impact of the missing data for continuous outcomes, the two following analyses will be performed:

1. “Best-worst-case” scenario: It will be assumed that all participants lost to follow-up in the experimental group had mean (from patients with follow-up) + 2 × SD; and all those with missing outcomes in the control group had mean (from patients with follow-up) − 2 × SD [70].
2. “Worst-best-case” scenario: It will be assumed that all participants lost to follow-up in the experimental group had mean (from patients with follow-up) − 2 × SD; and all those with missing outcomes in the control group had mean (from patients with follow-up) + 2 × SD [70].
  To assess the potential impact of missing SDs for continuous outcomes, the following sensitivity analyses will be performed: where SDs are missing and not possible to calculate, SDs will be imputed from trials with similar populations and low risk of bias. If no such trials can be found, SDs will be imputed from the trials with a similar population. As the final option, SDs will be imputed from all the trials.

### Summary of findings

We will present our findings in tables categorising reviews into (1) systematic reviews according to PRISMA with low risk of bias assessed with ROBIS, (2) systematic reviews according to PRISMA with high risk of bias assessed with ROBIS, and (3) non-systematic reviews according to PRISMA.

We will use the GRADE system [94] to assess the quality of the body of evidence identified from systematic reviews with low risk of bias associated with each of the primary outcomes (all-cause mortality, serious adverse events, resolution of delirium (treatment)/incidence (preventon)) and secondary outcomes (quality of life, non-serious adverse events, cognitive function) by constructing summary of findings (SoF) tables using the GRADE software [95]. For each primary and secondary outcome, firstly, we will present the summary of findings in randomised clinical trials with overall low risk of bias and, secondarily, the results in all the trials.

The GRADE approach appraises the quality of a body of evidence based on the extent to which one can be confident that an estimate of effect or association reflects the item being assessed. The quality measure of a body of evidence considers within the study risk of bias, the directness of the evidence, heterogeneity of the data, precision of effect estimates [70], and risk of publication bias.

We will include all risk of bias in the SOF table and then downgrade the quality of the evidence to take the bias into account.

## Discussion

To our knowledge, no overview summarising the best available evidence of pharmacological interventions against delirium in the ICU has been published. Our systematic and comprehensive literature search is targeted to identify all published reviews and meta-analyses, and we aim to summarise the evidence based primarily on systematic reviews with low risk of bias. We define systematic reviews according to PRISMA [63] and low risk of bias according to ROBIS. This choice may limit the number of systematic reviews on which to base our conclusions, but at the same time, this is a strength as we are thus purporting only the best available evidence. The result of our review will define the need for a new high quality systematic review.

A preliminary search identified several reviews [28, 96–105] investigating the effects of pharmacological interventions for the management and prevention of delirium in the critically ill, of which some reveals conflicting results. E.g. Lacasse et al. [96] suggests that antipsychotic drugs are efficacious and safe for the management of delirium, while Serafim et al. [105] did not find an association between pharmacologic management and delirium duration. However, Serafim et al. [105] do suggest antipsychotics for surgical ICU patients and dexmedetomidine for mechanically ventilated patients as a preventive strategy.

Despite the conflicting and inconclusive results in supporting the use of pharmacological agents in the management of delirium, antipsychotics and, in particular, haloperidol remains the intervention recommended as drugs of first choice, even though the latest guideline states that no published evidence is in support of managing delirious critically ill patients, nor for prevention of delirium, with haloperidol [21].

We consider it important to perform an overview of reviews summarising systematically and critically the available evidence from reviews of randomised clinical trials on pharmacological interventions in managing and preventing delirium in the ICU patients. Based on this overview of reviews, it will be possible to update or to design a high quality systematic review and, subsequently, a high quality randomised clinical trial, of the most promising candidate for pharmacological intervention for managing (or preventing) delirium in the ICU patient. Identifying the most effective intervention for delirium in critically ill patients will benefit patients, healthcare systems, and healthcare economy throughout the world.

## Additional files

Additional file 1: Preferred Reporting Items for Systematic Reviews and Meta-analysis Protocols (PRISMA-P) 2015 checklist: recommended items to address in a systematic review protocol. (DOCX 28 kb)
Additional file 2: Search strategies for Cochrane Library, MEDLINE (OvidSP), EMBASE (OvidSP), Science Citation Index (web of science), BIOSIS Citation Index (web of science), Cumulative Index to Nursing & Allied Health Literature (CINAHL), Latin American Caribbean Health Sciences Literature (LILACS) and Allied and Complementary Medicine Database (AMED). (DOCX 30 kb)
Additional file 3: Data extraction form. (DOCX 25 kb)

## Abbreviations

CAM-ICU: Confusion Assessment Method for the ICU
ICDSC: Intensive Care Delirium Screening Checklist
ICU: Intensive Care Unit
PRISMA: Preferred Reporting Items for Systematic Reviews and Meta-Analyses
