# Supplementary material for: Pharmacological interventions for delirium in intensive care patients: a protocol for an overview of reviews
Source: Syst Rev. 2016 Dec 7;5:211. doi: 10.1186/s13643-016-0391-5 (PMC5142129; doi:10.1186/s13643-016-0391-5)
Supplement: Additional file 2: — Search strategies for Cochrane Library, MEDLINE (OvidSP), EMBASE (OvidSP), Science Citation Index (web of science), BIOSIS Citation Index (web of science), Cumulative Index to Nursing & Allied Health Literature (CINAHL), Latin American Caribbean Health Sciences Literature (LILACS) and Allied and Complementary Medicine Database (AMED). (DOCX 30 kb) [file 13643_2016_391_MOESM2_ESM.docx]

**Additional file 2**

**Search strategy for Cochrane Library**

#1 MeSH descriptor: [Antipsychotic Agents] explode all trees
#2 MeSH descriptor: [Hypnotics and Sedatives] explode all trees
#3 MeSH descriptor: [Benzodiazepines] explode all trees
#4 MeSH descriptor: [Analgesics, Opioid] explode all trees
#5 MeSH descriptor: [Melatonin] explode all trees
#6 (medication or drug* or agent* or pharmacologic* or antipsychotic* or sedative* or opioid* or benzodiazepin*or aripiprazole or clozapine or haloperidol or olanzapine or quetiapine or risperidone or ziprasidone or dexmedetomidine or clonidine or cholinesterase inhibitor or rivastigmine or donepezil or melatonin* or ketamine):ti,ab,kw
#7 (#1 or #2 or #3 or #4 or #5 or #6)
#8 MeSH descriptor: [Delirium] explode all trees
#9 (delirium* or (acut* near (brain and dysfunction))):ti,ab,kw
#10 (#8 or #9)
#11 MeSH descriptor: [Critical Care] explode all trees
#12 MeSH descriptor: [Intensive Care Units] explode all trees
#13 MeSH descriptor: [Heart Arrest] explode all trees
#14 MeSH descriptor: [Myocardial Infarction] explode all trees
#15 MeSH descriptor: [Shock] explode all trees
#16 MeSH descriptor: [Craniocerebral Trauma] explode all trees
#17 MeSH descriptor: [Stroke] explode all trees
#18 MeSH descriptor: [Sepsis] explode all trees
#19 MeSH descriptor: [Shock, Septic] explode all trees
#20 exp Acute Disease/su [Surgery]
#21 exp Abdomen, Acute/su [Surgery]
#22 exp Hip Fractures/su [Surgery]
#23 (((intensive or critical*) near/3 (care or unit or department* or ill*)) or ICU):ti,ab,kw
#24 (cardiac arrest or cardiac failure or CPR or heart arrest or heart failure or myocardial infarct* or shock or (traumatic brain injury or TBI or head trauma) or (stroke or intracranial bleeding or intracranial hemorrhage) or (sepsis or septic shock)):ti,ab,kw
#25 ((acute or emergency or urgent or trauma) near3 (surgery or operation or resection or section)):ti,ab,kw
#26 (#11 or #12 or #13 or #14 or #15 or #16 or #17 or #18 or #19 or #20 or #21 or #22 or #23 or #24 or #25)
#27 (#7 and #10 and #26)

**Search strategy for MEDLINE (OvidSP)**

1. exp Antipsychotic Agents/
2. exp "Hypnotics and Sedatives"/
3. exp Benzodiazepines/
4. exp Analgesics, Opioid/
5. exp Melatonin/
6. (medication or drug* or agent* or pharmacologic* or antipsychotic* or sedative* or opioid* or benzodiazepin*or aripiprazole or clozapine or haloperidol or olanzapine or quetiapine or risperidone or ziprasidone or dexmedetomidine or clonidine or cholinesterase inhibitor or rivastigmine or donepezil or melatonin* or ketamine).tw.
7. 1 or 2 or 3 or 4 or 5 or 6
8. exp Delirium/
9. (delirium* or (acut* adj (brain and dysfunction))).tw.
10. 8 or 9
11. exp Critical Care/
12. exp Intensive-Care-Units/
13. exp Heart Arrest/
14. exp Myocardial Infarction/
15. exp Shock/
16. exp Craniocerebral Trauma/
17. exp Stroke/
18. exp Sepsis/
19. exp Shock, Septic/
20. exp Acute Disease/su [Surgery]
21. exp Abdomen, Acute/su [Surgery]
22. exp Hip Fractures/su [Surgery]
23. (((intensive or critical*) adj3 (care or unit or department* or ill*)) or ICU).tw.
24. (cardiac arrest or cardiac failure or CPR or heart arrest or heart failure or myocardial infarct* or shock or (traumatic brain injury or TBI or head trauma) or (stroke or intracranial bleeding or intracranial hemorrhage) or (sepsis or septic shock)).tw.
25. (acute or emergency or urgent or trauma) adj3 (surgery or operation or resection or section).tw.
26. 11 or 12 or 13 or 14 or 15 or 16 or 17 or 18 or 19 or 20 or 21
27. 7 and 10 and 26

**Search strategy for EMBASE (OvidSP)**

1. *neuroleptic agent/
2. *sedative agent/
3. *opiate/
4. *benzodiazepine derivative/
5. *cholinesterase inhibitor/
6. *melatonin/
7. (medication or drug* or agent* or pharmacologic* or antipsychotic* or sedative* or opioid* or benzodiazepin*or aripiprazole or clozapine or haloperidol or olanzapine or quetiapine or risperidone or ziprasidone or dexmedetomidine or clonidine or cholinesterase inhibitor or rivastigmine or donepezil or melatonin* or ketamine).tw.
8. 1 or 2 or 3 or 4 or 5 or 6 or 7
9. *delirium/
10. (delirium* or (acut* adj (brain and dysfunction))).tw.
11. 9 or 10
12. *critical illness/
13. *intensive care/
14. *intensive care unit/
15. *heart arrest/
16. *heart infarction/
17. *shock/
18. *traumatic brain injury/
19. *cerebrovascular accident/
20. *sepsis/
21. *septic shock/
22. *acute disease/su [Surgery]
23. *acute abdomen/su [Surgery]
24. *emergency surgery/
25. *hip fracture/su [Surgery]
26. (((intensive or critical*) adj3 (care or unit or department* or ill*) or ICU).tw.
27. (cardiac arrest or cardiac failure or CPR or heart arrest or heart failure or myocardial infarct* or shock or (traumatic brain injury or TBI or head trauma) or (stroke or intracranial bleeding or intracranial hemorrhage) or (sepsis or septic shock)).tw.
28. (acute or emergency or urgent or trauma) adj3 (surgery or operation or resection or section).tw.
29. 12 or 13 or 14 or 15 or 16 or 17 or 18 or 19 or 20 or 21 or 22 or 23 or 24 or 25 or 26 or 27 or 28
30. 8 and 11 and 29

**Search strategy for Science Citation Index–Expanded**

#1 TOPIC: ((medication or drug* or agent* or pharmacologic* or antipsychotic* or sedative* or opioid* or benzodiazepin*or aripiprazole or clozapine or haloperidol or olanzapine or quetiapine or risperidone or ziprasidone or dexmedetomidine or clonidine or cholinesterase inhibitor or rivastigmine or donepezil or melatonin* or ketamine))
#2 TOPIC: ((delirium* or (acut* near3 (brain and dysfunction))))
#3 TOPIC: ((((intensive or critical*) near3 (care or unit or department* or ill*)) or ICU))
#4 TOPIC: ((cardiac arrest or cardiac failure or CPR or heart arrest or heart failure or myocardial infarct* or shock or (traumatic brain injury or TBI or head trauma) or (stroke or intracranial bleeding or intracranial hemorrhage) or (sepsis or septic shock)))
#5 TOPIC: ((acute or emergency or urgent or trauma) near3 (surgery or operation or resection or section))
#6 (#3 OR #4 OR #5)
#7 (#1 AND #2 AND #6)

**Search strategy for BIOSIS Previews**

#1 TOPIC: ((medication or drug* or agent* or pharmacologic* or antipsychotic* or sedative* or opioid* or benzodiazepin*or aripiprazole or clozapine or haloperidol or olanzapine or quetiapine or risperidone or ziprasidone or dexmedetomidine or clonidine or cholinesterase inhibitor or rivastigmine or donepezil or melatonin* or ketamine))
#2 TOPIC: ((delirium* or (acut* near3 (brain and dysfunction))))
#3 TOPIC: ((((intensive or critical*) near3 (care or unit or department* or ill*)) or ICU))
#4 TOPIC: ((cardiac arrest or cardiac failure or CPR or heart arrest or heart failure or myocardial infarct* or shock or (traumatic brain injury or TBI or head trauma) or (stroke or intracranial bleeding or intracranial hemorrhage) or (sepsis or septic shock)))
#5 TOPIC: ((acute or emergency or urgent or trauma) near3 (surgery or operation or resection or section))
#6 (#3 OR #4 OR #5)
#7 (#1 AND #2 AND #6)

**Search strategy for Cumulative Index to Nursing & Allied Health Literature (CINAHL)**

S1 MJ Antipsychotic Agents
S2 MJ hypnotics and sedatives
S3 MM Antianxiety Agents, Benzodiazepine
S4 MJ Analgesics, Opioid
S5 MJ Melatonin
S6 AB (medication or drug* or agent* or pharmacologic* or antipsychotic* or sedative* or opioid* or benzodiazepin*or aripiprazole or clozapine or haloperidol or olanzapine or quetiapine or risperidone or ziprasidone or dexmedetomidine or clonidine or cholinesterase inhibitor or rivastigmine or donepezil or melatonin* or ketamine)
S7 (S1 OR S2 OR S3 OR S4 OR S5 OR S6)
S8 MJ Delirium
S9 AB (delirium* or (acut* and (brain and dysfunction)))
S10 (S8 OR S9)
S11 MJ critical care
S12 MJ Intensive Care Units
S13 MJ Heart Arrest
S14 MJ Myocardial Infarction
S15 MJ Shock
S16 MJ brain injuries
S17 MJ stroke
S18 MJ sepsis
S19 MJ septic shock

S20 MJ acute surgery

S21 (MH "Acute Disease/SU")

S22 (MH "Hip Fractures/SU")
S23 AB (((intensive or critical*) and (care or unit or department* or ill*)) or ICU)
S24 AB (cardiac arrest or cardiac failure or CPR or heart arrest or heart failure or myocardial infarct* or shock or (traumatic brain injury or TBI or head trauma) or (stroke or intracranial bleeding or intracranial hemorrhage) or (sepsis or septic shock))
S25 AB ((acute or emergency or urgent or trauma) and (surgery or operation or resection or section))
S26 (S11 OR S12 OR S13 OR S14 OR S15 OR S16 OR S17 OR S18 OR S19 OR S20 OR S21 OR S22 OR S23 OR S24 OR S25)
S27 (S7 AND S10 AND S26)

**Search strategy for Latin American Caribbean Health Sciences Literature (LILACS)**

(tw:((medication or drug* or agent* or pharmacologic* or antipsychotic* or sedative* or opioid* or benzodiazepin*or aripiprazole or clozapine or haloperidol or olanzapine or quetiapine or risperidone or ziprasidone or dexmedetomidine or clonidine or cholinesterase inhibitor or rivastigmine or donepezil or melatonin* or ketamine))) AND (tw:((delirium* or (acut* and (brain and dysfunction))))) AND (tw:((((intensive or critical*) and (care or unit or department* or ill*)) or ICU) OR ((cardiac arrest or cardiac failure or CPR or heart arrest or heart failure or myocardial infarct* or shock or (traumatic brain injury or TBI or head trauma) or (stroke or intracranial bleeding or intracranial hemorrhage) or (sepsis or septic shock) or ((acute or emergency or urgent or trauma) and (surgery or operation or resection or section))))))

**Search strategy for Allied and Complementary Medicine Database (AMED)**

S1 TX (medication or drug* or agent* or pharmacologic* or antipsychotic* or sedative* or opioid* or benzodiazepin*or aripiprazole or clozapine or haloperidol or olanzapine or quetiapine or risperidone or ziprasidone or dexmedetomidine or clonidine or cholinesterase inhibitor or rivastigmine or donepezil or melatonin* or ketamine)
S2 TX delirium* OR TX ( (acut* and (brain and dysfunction)) )
S3 TX ( (((intensive or critical*) and (care or unit or department* or ill*)) or ICU) ) OR TX ( (cardiac arrest or cardiac failure or CPR or heart arrest or heart failure or myocardial infarct* or shock or (traumatic brain injury or TBI or head trauma) or (stroke or intracranial bleeding or intracranial hemorrhage) or (sepsis or septic shock)) or ((acute or emergency or urgent or trauma) and (surgery or operation or resection or section)) )
S4 (S1 and S2 and S3)
