# Supplementary material for: Pharmacological interventions for delirium in intensive care patients: a protocol for an overview of reviews
Source: Syst Rev. 2016 Dec 7;5:211. doi: 10.1186/s13643-016-0391-5 (PMC5142129; doi:10.1186/s13643-016-0391-5)
Supplement: Additional file 3: — Data extraction form. (DOCX 25 kb) [file 13643_2016_391_MOESM3_ESM.docx]

**Additional file 3**

**Data extraction form**

**REVIEW IDENTIFICATION**

| **Author** |  |
| --- | --- |
| **Year** |  |
| **Title** |  |

**REVIEW ELIGIBILITY**

| **Review** | | | **Relevant participants** | | | **Relevant intervention** | | | **Relevant outcomes** | | |
| --- | --- | --- | --- | --- | --- | --- | --- | --- | --- | --- | --- |
| Yes | No | Unclear | Yes | No | Unclear | Yes | No | Unclear | Yes | No | Unclear |

DO NOT PROCEED IF ANY OF THE ABOVE ANSWERS IS ‘NO’

| **Include** | **Exclude**  Record reason for exclusion |
| --- | --- |
|  |  |

**PRISMA CHECKLIST**

| **Section/Topic** | **#** | **Checklist item** | **Reported on page #** |
| --- | --- | --- | --- |
| **TITLE** | | | |
| Title | 1 | Identify the report as a systematic review, meta-analysis, or both |  |
| **ABSTRACT** | | | |
| Structured summary | 2 | Provide a structured summary including, as applicable: background; objectives; data sources; study eligibility criteria, participants, and interventions; study appraisal and synthesis methods; results; limitations; conclusions and implications of key findings; systematic review registration number |  |
| **INTRODUCTION** | | | |
| Rationale | 3 | Describe the rationale for the review in the context of what is already known |  |
| Objectives | 4 | Provide an explicit statement of questions being addressed with reference to participants, interventions, comparisons, outcomes, and study design (PICOS) |  |
| **METHODS** | | | |
| Protocol and registration | 5 | Indicate if a review protocol exists, if and where it can be accessed (e.g., Web address), and, if available, provide registration information including registration number. |  |
| Eligibility criteria | 6 | Specify study characteristics (e.g., PICOS, length of follow-up) and report characteristics (e.g., years considered, language, publication status) used as criteria for eligibility, giving rationale |  |
| Information sources | 7 | Describe all information sources (e.g., databases with dates of coverage, contact with study authors to identify additional studies) in the search and date last searched |  |
| Search | 8 | Present full electronic search strategy for at least one database, including any limits used, such that it could be repeated |  |
| Study selection | 9 | State the process for selecting studies (i.e., screening, eligibility, included in systematic review, and, if applicable, included in the meta-analysis) |  |
| Data collection process | 10 | Describe method of data extraction from reports (e.g., piloted forms, independently, in duplicate) and any processes for obtaining and confirming data from investigators |  |
| Data items | 11 | List and define all variables for which data were sought (e.g., PICOS, funding sources) and any assumptions and simplifications made |  |
| Risk of bias in individual studies | 12 | Describe methods used for assessing risk of bias of individual studies (including specification of whether this was done at the study or outcome level), and how this information is to be used in any data synthesis |  |
| Summary measures | 13 | State the principal summary measures (e.g., risk ratio, difference in means) |  |
| Synthesis of results | 14 | Describe the methods of handling data and combining results of studies, if done, including measures of consistency (e.g., I2) for each meta-analysis |  |
| Risk of bias across studies | 15 | Specify any assessment of risk of bias that may affect the cumulative evidence (e.g., publication bias, selective reporting within studies) |  |
| Additional analyses | 16 | Describe methods of additional analyses (e.g., sensitivity or subgroup analyses, meta-regression), if done, indicating which were pre-specified |  |
| **RESULTS** | | | |
| Study selection | 17 | Give numbers of studies screened, assessed for eligibility, and included in the review, with reasons for exclusions at each stage, ideally with a flow diagram |  |
| Study characteristics | 18 | For each study, present characteristics for which data were extracted (e.g., study size, PICOS, follow-up period) and provide the citations |  |
| Risk of bias within studies | 19 | Present data on risk of bias of each study and, if available, any outcome level assessment (see item 12) |  |
| Results of individual studies | 20 | For all outcomes considered (benefits or harms), present, for each study: (a) simple summary data for each intervention group (b) effect estimates and confidence intervals, ideally with a forest plot |  |
| Synthesis of results | 21 | Present results of each meta-analysis done, including confidence intervals and measures of consistency |  |
| Risk of bias across studies | 22 | Present results of any assessment of risk of bias across studies (see Item 15) |  |
| Additional analysis | 23 | Give results of additional analyses, if done (e.g., sensitivity or subgroup analyses, meta-regression [see Item 16]) |  |
| **DISCUSSION** | | | |
| Summary of evidence | 24 | Summarize the main findings including the strength of evidence for each main outcome; consider their relevance to key groups (e.g., healthcare providers, users, and policy makers) |  |
| Limitations | 25 | Discuss limitations at study and outcome level (e.g., risk of bias), and at review-level (e.g., incomplete retrieval of identified research, reporting bias) |  |
| Conclusions | 26 | Provide a general interpretation of the results in the context of other evidence, and implications for future research |  |
| **FUNDING** | | | |
| Funding | 27 | Describe sources of funding for the systematic review and other support (e.g., supply of data); role of funders for the systematic review |  |

**REVIEW ASSESSED TO BE SYSTEMATIC ACCORDING TO PRISMA**

| **YES** | **NO** |
| --- | --- |

**RISK OF BIAS ASSESSMENT OF SYSTEMATIC REVIEWS USING ROBIS**

**Identifying concerns with the review**

| **DOMAIN 1: STUDY ELIGIBILITY CRITERIA** | | |
| --- | --- | --- |
| Describe the study eligibility criteria, any restrictions on eligibility and whether there was evidence that objectives and eligibility criteria were pre-specified: | | |
| 1.1 | Did the review adhere to pre-defined objectives and eligibility criteria? | Y/PY/PN/N/NI |
| 1.2 | Were the eligibility criteria appropriate for the review question? | Y/PY/PN/N/NI |
| 1.3 | Were eligibility criteria unambiguous? | Y/PY/PN/N/NI |
| 1.4 | Were all restrictions in eligibility criteria based on study characteristics appropriate (e.g. date, sample size, study quality, outcomes measured)? | Y/PY/PN/N/NI |
| 1.5 | Were any restrictions in eligibility criteria based on sources of information appropriate (e.g. publication status or format, language, availability of data)? | Y/PY/PN/N/NI |
| Concerns regarding specification of study eligibility criteria  Rationale for concern: | | LOW/HIGH/UNCLEAR |

| **DOMAIN 2: IDENTIFICATION AND SELECTION OF STUDIES** | | |
| --- | --- | --- |
| Describe methods of study identification and selection (e.g. number of reviewers involved): | | |
| 2.1 | Did the search include an appropriate range of databases/electronic sources for published and unpublished reports? | Y/PY/PN/N/NI |
| 2.2 | Were methods additional to database searching used to identify relevant reports? | Y/PY/PN/N/NI |
| 2.3 | Were the terms and structure of the search strategy likely to retrieve as many eligible studies as possible? | Y/PY/PN/N/NI |
| 2.4 | Were restrictions based on date, publication format, or language appropriate? | Y/PY/PN/N/NI |
| 2.5 | Were efforts made to minimise error in selection of studies? | Y/PY/PN/N/NI |
| Concerns regarding methods used to identify and/or select studies  Rationale for concern: | | LOW/HIGH/UNCLEAR |

| **DOMAIN 3: DATA COLLECTION AND STUDY APPRAISAL** | | |
| --- | --- | --- |
| Describe methods of data collection, what data were extracted from studies or collected through other means, how risk of bias was assessed (e.g. number of reviewers involved) and the tool used to assess risk of bias: | | |
| 3.1 | Were efforts made to minimise error in data collection? | Y/PY/PN/N/NI |
| 3.2 | Were sufficient study characteristics available for both review authors and readers to be able to interpret the results? | Y/PY/PN/N/NI |
| 3.3 | Were all relevant study results collected for use in the synthesis? | Y/PY/PN/N/NI |
| 3.4 | Was risk of bias (or methodological quality) formally assessed using appropriate criteria? | Y/PY/PN/N/NI |
| 3.5 | Were efforts made to minimise error in risk of bias assessment? | Y/PY/PN/N/NI |
| Concerns regarding methods used to collect data and appraise studies  Rationale for concern: | | LOW/HIGH/UNCLEAR |

| **DOMAIN 4: SYNTHESIS AND FINDINGS** | | |
| --- | --- | --- |
| Describe synthesis methods: | | |
| 4.1 | Did the synthesis include all studies that it should? | Y/PY/PN/N/NI |
| 4.2 | Were all pre-defined analyses reported or departures explained? | Y/PY/PN/N/NI |
| 4.3 | Was the synthesis appropriate given the nature and similarity in the research questions, study designs and outcomes across included studies? | Y/PY/PN/N/NI |
| 4.4 | Was between-study variation (heterogeneity) minimal or addressed in the synthesis? | Y/PY/PN/N/NI |
| 4.5 | Were the findings robust, e.g. as demonstrated through funnel plot or sensitivity analyses? | Y/PY/PN/N/NI |
| 4.6 | Were biases in primary studies minimal or addressed in the synthesis? | Y/PY/PN/N/NI |
| Concerns regarding the synthesis and findings  Rationale for concern: | | LOW/HIGH/UNCLEAR |

Y=YES, PY=PROBABLY YES, PN=PROBABLY NO, N=NO, NI=NO INFORMATION

**Judging risk of bias**

Summarize the concerns identified during ‘Identifying concerns with the review’ assessment:

| Domain | Concern | Rationale for concern |
| --- | --- | --- |
| 1. Concerns regarding specification of study eligibility criteria |  |  |
| 2. Concerns regarding methods used to identify and/or select studies |  |  |
| 3. Concerns regarding used to collect data and appraise studies |  |  |
| 4. Concerns regarding the synthesis and findings |  |  |

**RISK OF BIAS IN THE SYSTEMATIC REVIEW**

| Describe whether conclusions were supported by the evidence: | | |
| --- | --- | --- |
| A | Did the interpretation of findings address all of the concerns identified in Domains 1 to 4? | Y/PY/PN/N/NI |
| B | Was the relevance of identified studies to the review's research question appropriately considered? | Y/PY/PN/N/NI |
| C | Did the reviewers avoid emphasizing results on the basis of their statistical significance? | Y/PY/PN/N/NI |
| Risk of bias in the systematic review | | RISK: LOW/HIGH/UNCLEAR |

Y=YES, PY=PROBABLY YES, PN=PROBABLY NO, N=NO, NI=NO INFORMATION

**CHARACTERISTICS OF SYSTEMATIC REVIEW**

| Number of trials included |  |
| --- | --- |
| Number of participants included |  |
| ICU population (e.g. medical) |  |
| Diagnostic criteria of delirium |  |
| Type of pharmacological agent(s) included |  |
| Primary and secondary outcomes |  |
| Results on primary and secondary outcomes |  |
| Type of meta-analytic and sequential analysis used |  |
| Authors’ conclusion/recommendation |  |

**CHARACTERISTICS OF NON-SYSTEMATIC REVIEW**

| Intervention | Prevention  Management |
| --- | --- |
| Recommendation |  |
